# Mechanisms of lipid homeostasis in the *Coxiella* Containing Vacuole

**DOI:** 10.1042/BST20240899

**Published:** 2025-01-22

**Authors:** Rajendra K. Angara, Peyton E. Van Winkle, Stacey D. Gilk

**Affiliations:** Department of Pathology, Microbiology, and Immunology, University of Nebraska Medical Center, Omaha, Nebraska, U.S.A

**Keywords:** cholesterol, *Coxiella burnetti*, *Coxiella *Containing Vacuole, lipid droplets, lipids, phosphatidylinositol phosphates (PIPs)

## Abstract

*Coxiella burnetii*, the causative agent of human Q fever, is an obligate intracellular bacterial pathogen that replicates in a large, membrane-bound vacuole known as the *Coxiella* Containing Vacuole (CCV). The CCV is a unique, phagolysosome-derived vacuole with a sterol-rich membrane containing host and bacterial proteins. The CCV membrane itself serves as a barrier to protect the bacteria from the host’s innate immune response, and the lipid and protein content directly influence both the CCV luminal environment and interactions between the CCV and host trafficking pathways. CCV membrane cholesterol is critical in regulating CCV pH, while CCV phosphatidylinositol phosphate species influence CCV fusion events and membrane dynamics. *C. burnetii* proteins directly target host lipid metabolism to regulate CCV membrane content and generate a source of lipids that support bacterial replication or influence the innate immune response. This review provides an overview of the diverse repertoire of lipids involved in CCV formation and maintenance, highlighting the pathogen-driven strategies to modify host lipid homeostasis.

## Introduction

Obligate intracellular bacterial pathogens survive in diverse intracellular niches. For vacuole-bound pathogens such as *Coxiella burnetii, Anaplasma phagocytophilum,* and *Chlamydia* species, the formation and maintenance of bacteria-containing vacuoles are essential for pathogenesis, with each pathogen employing unique strategies to manipulate the host cell to form these vacuoles. *C. burnetii,* the causative agent of Q fever, hijacks the host endocytic trafficking to create the specialized *Coxiella* Containing Vacuole (CCV) [1]. *C. burnetii,* which can be found in lipid droplet-rich foamy macrophages of infected endocarditis patients [2], utilizes host lipids to establish successful infection and replication inside the CCV. Host cell lipids play multiple roles as mediators of immune defense and essential resources for bacterial structural integrity and energy. This review outlines the key roles of lipids in CCV formation and maintenance and details *C. burnetii*-driven mechanisms regulating host lipid homeostasis.

### Lipid requirements for the *C. burnetii* intracellular developmental life cycle

The *C. burnetti* biphasic developmental life cycle consists of a small cell variant (SCV) and a large cell variant (LCV), with both forms capable of infecting host cells. The SCV is metabolically dormant and structurally resilient, enabling *C. burnetii* to persist in the environment. Within 8 hours of entering the host cell, the SCV differentiates to a metabolically active LCV, which replicates in the CCV lumen. Around 6 days post-infection, the bacteria enter the stationary phase, and LCVs differentiate back to SCVs [3]. SCV to LCV transition is characterized by distinct changes in the membrane lipid profile. Phosphatidylethanolamine (PE), phosphatidylglycerol (PG), and cardiolipin are high in the LCV, while SCVs contain cardiolipin, lyso-PE, and free fatty acids, with less PE and PG [4]. Bacterial replication requires phospholipids to form the bacterial membrane, and the *C. burnetii* genome contains predicted enzymes to generate PE, PG, phosphatidylserine (PS), and cardiolipin [5,6].

### Biogenesis of the *Coxiella* Containing Vacuole

*C. burnetii* intracellular survival requires the CCV, a large, dynamic, and complex membrane-bound organelle. CCV formation begins with *C. burnetii* phagocytosis, during which the bacterium binds to surface receptors, including the lipid raft-associated integrin α_v_β_3_, to initiate actin cytoskeleton remodeling and membrane rearrangement [7–9]. Plasma membrane lipid rafts composed of cholesterol, glycosphingolipids, and glycerophospholipids are critical for *C. burnetii* internalization. Cholesterol is implicated in this process*,* as cholesterol-free cells inefficiently internalize *C. burnetii,* most likely due to defective lipid raft signaling [8]. Upon host cell entry, the *C. burnetii*-containing phagosome matures through the endocytic pathway to a phagolysosome [10,11], where the acidic pH is required to activate *C. burnetii* metabolism and trigger SCV to LCV differentiation [12]. Within 24 hours, the pH elevates and stabilizes at ~pH 5.2, and the bacteria undergo one or two rounds of replication in a small and tight-fitting vacuole [13,14]. Around 24–48 hours post-infection, fusion between the CCV and host endosomes, lysosomes, and autophagosomes leads to rapid CCV expansion [15]. The mature CCV is a large, moderately acidic (pH~5.2) vacuole that supports the exponential phase of bacterial replication, with a bacterial doubling time of 10–12 hours [13,16].

From host cell entry to CCV formation and maintenance, *C. burnetii* intracellular survival requires the Type 4B Secretion System (T4BSS), which secretes ~150 effector proteins across the CCV membrane and into the host cell cytoplasm [17,18]. T4BSS effector proteins manipulate multiple host pathways, including vesicle trafficking, lipid metabolism, autophagy, and apoptosis [19]. Many of these pathways are central to the biology and function of the CCV, particularly lipids, which serve as building blocks of the CCV membrane, signaling molecules for membrane fusion events, immune response mediators, and potential energy sources for replicating bacteria [20,21].

### CCV membrane lipids and their influence on CCV biology

While the complete CCV membrane lipid composition is unknown, PS, lysobisphosphatidic acid (LBPA), cholesterol, and phosphatidylinositol phosphates (PIPs) are significant components of the CCV membrane [22–24]. LBPA is enriched in the CCV in a T4BSS-dependent manner, while PS is passively acquired by the CCV [23]. LBPA, found primarily on the inner membranes of late endosomes, is a unique phospholipid essential for endosomal function and dynamics [25]. In particular, LBPA helps organize the intraluminal vesicles within multivesicular bodies (MVBs), a subtype of late endosomes, by promoting the inward budding and sorting of cargo into these vesicles.

PIPs are important signaling lipids with organelle-specific localization and a broader distribution than LBPA [26]. PIPs are pivotal during phagosome maturation by coordinating proteins involved in signal transduction and vesicular trafficking. PIP-binding proteins contain domains specific for different PIP species and include FYVE (Fab1-YotB-Vac1-EEA1), pleckstrin homology (PH), and phox homology (PX) domains [27]; these PIP-binding proteins regulate fusion events required for endosomal maturation. Phosphoinositide kinases and phosphatases control PIP interconversion and influence phagosomal PIP species, which regulate vesicular maturation. The CCV contains a subset of PIP species, primarily PI(3)P and PI(4)P [23]. Interestingly, PI(3,5)P_2_ is found on few CCVs until late infection (>6 days post-infection), when over 50% of CCVs contain PI(3,5)P_2_ [23]. CCV membrane PI(3)P likely supports CCV formation by recruiting host cell proteins involved in endosomal trafficking, fusion, and autophagy [23,24,28]. Additionally, CCV membrane PI(3)P serves as a signaling hub for recruiting specific bacterial T4BSS effectors that manipulate host processes, enabling the bacterium to control host lipid trafficking and promote vacuole expansion [24,28]. PI(4)P is typically associated with the Golgi but also plays a role in inter-organelle contact sites and may recruit host or bacterial proteins to the CCV membrane [26].

The CCV membrane is enriched for cholesterol and other sterol species [5,22,29,30]. In eukaryotic cells, cholesterol is transported by vesicular trafficking or sterol transfer proteins, particularly at inter-organelle membrane contact sites. Based on the importance of CCV fusion with the host endocytic pathway, vesicular trafficking presumably serves as the primary source of CCV cholesterol, although a role for sterol transfer proteins cannot be excluded. The role of cholesterol in the CCV membrane is not well-understood, but it may contribute to CCV membrane fluidity, regulate trafficking to the CCV, or serve as a possible mechanism to activate the T4BSS. The CCV membrane exhibits microdomains, indicated by staining for the lipid raft-associated protein Filipin1 and by actin patches at sites of CCV-endosome fusion; however, it remains uncertain whether these microdomains are cholesterol-rich [22,31]. It is clear, however, that CCV cholesterol directly influences the pH of the CCV lumen, where elevated CCV cholesterol leads to increased CCV acidification to pH<4.7 [29]. The CCV contains host lysosomal proteases and hydrolases, and cholesterol-induced acidification further activates CCV proteolytic activity and leads to bacterial degradation [29,30,32]. *C. burnetii* is particularly sensitive to drugs that elevate cholesterol in endosomes and lysosomes [22,29,33,34]. Interestingly, this sensitivity only occurs in the first 48 hours post-infection, suggesting that an established CCV may have protective mechanisms to prevent cholesterol toxicity or that the bacteria can resist increased acidification during the logarithmic phase of growth. While how CCV membrane cholesterol levels alters CCV pH is unknown, one plausible explanation is that cholesterol regulates ion transporters (e.g., vATPase) found on the CCV membrane [35]. As detailed below, *C. burnetii* uses a variety of strategies to regulate CCV cholesterol levels, including enzymatic modification of cholesterol and membrane contact sites to transfer cholesterol from the CCV to the host endoplasmic reticulum (ER) [14,32].

### *C. burnetii* sterol-modifying enzymes

The *C. burnetii* genome lacks genes for cholesterol biosynthesis or catabolism, and no cholesterol or other sterols have been detected in the bacterial membrane [6,8]. Additionally, *C. burnetii* axenic growth does not require cholesterol [29]. Together, these findings indicate that *C. burnetii* does not have a direct requirement for cholesterol. However, the *C. burnetii* genome encodes two eukaryotic-like sterol reductases, CBU1158 (a putative ∆7 sterol reductase) and CBU1206/Stmp1 (a putative ∆24 sterol reductase) [6,36,37]. Although the enzymatic function of CBU1158 is unknown, CBU1206 encodes an active enzyme, Sterol Modifying Protein-1 (Stmp1), which rescues a mutant in the yeast homolog [30,37]. A *C. burnetii* Δ*stmp1* mutant has increased CCV membrane cholesterol, indicating that Stmp1 decreases CCV cholesterol through enzymatic modification [30]. While the exact enzymatic mechanism is unknown, *C. burnetii* Δ*stmp1*-infected cells accumulate the cholesterol metabolite 25-hydroxycholesterol (25-HC), a critical sterol derivative that regulates sterol homeostasis and the innate immune response [38–40]. Based on the exogenous treatment of infected cells, 25-HC directly affects CCV proteolytic activity and *C. burnetii* survival [30]. Indeed, 25-HC helps defend against bacterial infections such as *Listeria monocytogenes* and *Shigella flexneri* by reducing plasma membrane cholesterol, thus reducing the ability of the bacteria to invade adjacent cells [38,40]. Within host cells, 25-HC-mediated immune responses protect against highly pathogenic viruses such as human immunodeficiency virus (HIV), Ebola virus, Zika virus, and SARS-CoV-2 [40–42]. Considering the pivotal role of 25-HC in sterol homeostasis and innate immunity, further investigation is necessary to elucidate the relationship between *C. burnetii* sterol reductases, 25-HC, and host sterol metabolism.

### *C. burnetii* regulates cholesterol transfer at CCV-ER membrane contact sites

**Figure 1:**
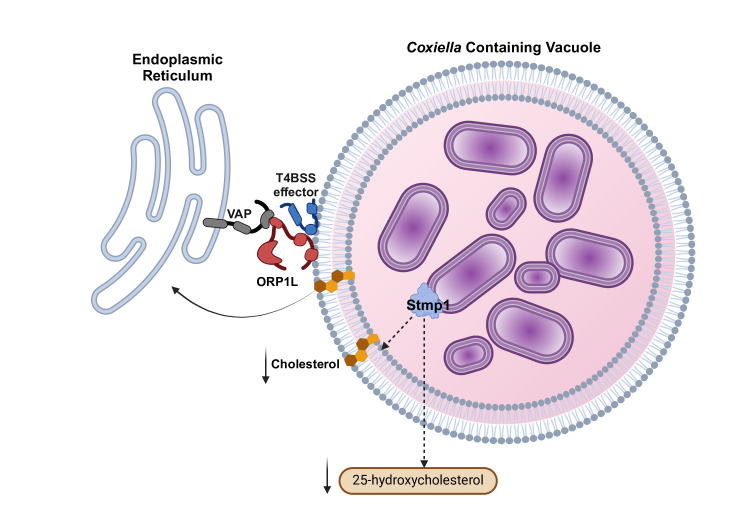
*C. burnetii* regulates cholesterol levels on the CCV membrane using ER-CCV membrane contact sites and sterol reductases. Through an as-yet-unknown effector, *C. burnetii* recruits the cholesterol transporter, ORP1L, to the CCV. At the CCV, ORP1L binds to the ER membrane protein VAPB, forming a CCV-ER membrane contact site to facilitate cholesterol transport from the CCV to the ER. The *C. burnetii* outer membrane protein, Stmp1, a sterol reductase, is critical for maintaining cholesterol levels on the CCV membrane. Loss of Stmp1 leads to the accumulation of cholesterol in the CCV membrane as well as higher host cell levels of the cholesterol metabolite 25-hydroxycholesterol. Created in BioRender. VanWinkle, P. (2024) https://BioRender.com/p57z559.

Membrane contact sites are unique regions where two membranes, typically of different organelles, are in close proximity (less than 30–40 nm) but do not fuse; these sites are locations for inter-organelle small molecule exchange. Membrane contact sites contain specialized proteins and protein complexes, including tether proteins mediating contact sites, regulatory proteins modulating contact site protein function, and lipid transport proteins facilitating lipid transfer between organelles [43]. Recently, the membrane contact site protein ORP1L (Oxysterol-Binding Protein-Related Protein 1 Long) was identified as a critical player during *C. burnetii* infection. ORP1L plays multiple roles in endosomal biology, including cholesterol-dependent endocytic trafficking along microtubules and cholesterol transport at membrane contact sites between late endosomes/lysosomes and the ER [44,45]. ORP1L contains several critical domains that dictate protein function, including N-terminal ankyrin repeats, an FFAT (two phenylalanines in an acidic tract) motif, and a C-terminal oxysterol binding domain (ORD). The N-terminal ankyrin repeats, known to bind the late endosome/lysosome protein Rab7, are necessary and sufficient for targeting ORP1L to the CCV [14]. However, ORP1L CCV localization is independent of Rab7, suggesting that additional protein–protein interactions are required [14]. Intriguingly, *C. burnetii* recruits ORP1L to the CCV membrane in a T4BSS-dependent manner, although the secreted effector protein(s) responsible for recruiting ORP1L to the CCV have yet to be identified [14,32] (Figure 1). The ORP1L FFAT motif binds members of the ER VAP (Vesicle-associated membrane protein-associated proteins) protein family to establish CCV-ER membrane contact sites. ORP1L-mediated CCV-ER membrane contact sites facilitate cholesterol trafficking from CCV to the ER and minimize cholesterol accumulation on the CCV membrane [32]. However, it is not clear whether ORP1L directly transfers cholesterol or recruits additional lipid transfer proteins to the CCV-ER membrane contact site. Interestingly, ORP1L is essential for *C. burnetii* growth in macrophages but not epithelial cells, which may indicate that ORP1L plays a more significant role in cholesterol homeostasis and membrane contact sites in macrophages [32].

Cholesterol transport by the export protein NPC1 may also influence CCV cholesterol levels, as suggested by poor *C. burnetii* growth in NPC1-deficient cells [33]. NPC1 is a late endosome/lysosomal membrane protein that exports cholesterol from late endosomes/lysosomes to the ER. However, whether NPC1 is directly or indirectly involved in ER-CCV contact sites needs further investigation. While NPC1 does not appear to facilitate membrane contact sites directly, NPC1-mediated cholesterol transport influences interactions between VAPB and the cholesterol transport protein OSBP at lysosome-ER membrane contact sites [46]. In addition to ORP1L, *C. burnetii* may recruit lipid transporters other than ORP1L to ER-CCV contact sites, given that lipid transporters facilitate efficient lipid exchange between organelle membranes, with the ER serving as a central hub for numerous tether proteins and lipid transport proteins. Beyond utilizing host tether proteins and lipid transport proteins, bacterial effector proteins may also directly mediate contact sites between the bacteria-containing vacuole and the host ER. For example, the obligate intracellular bacterium *Chlamydia trachomatis* expresses bacterial effector proteins that directly establish membrane contact sites between the bacteria-containing vacuole (known as an inclusion) and the host ER [47,48]. Therefore, it is possible that one or more *C. burnetii* effector protein(s) directly mediate ER-CCV membrane contact sites to facilitate the exchange of cholesterol and other lipids.

### *C. burnetii* alters lipid droplet metabolism

Lipid droplets (LDs) are dynamic cellular organelles that store neutral lipids such as triacylglycerols (TAG) and cholesterol esters (CE). Structurally, the LD core consists of hydrophobic lipids surrounded by a phospholipid monolayer embedded with proteins. LDs play a crucial role in energy metabolism, lipid homeostasis, and membrane synthesis, as well as contribute to lipid trafficking, signaling, and cellular stress responses through interactions with other organelles, such as the ER and mitochondria [49]. *C. burnetii* induces LDs in host cells in a T4BSS-dependent manner, and LD homeostasis is critical for *C. burnetii* survival and replication within the host cell [50]. Furthermore, *C. burnetii*-infected macrophages have increased expression of host proteins involved in LD homeostasis, including LD biogenesis (Acyl-CoA:cholesterol Transferase – ACAT), LD breakdown (Patatin-like Phospholipase Domain Containing Protein 2 – PNPLA2; Adipose Triglyceride Lipase – ATGL), and mobilization of LD-derived lipids (Fatty Acid Binding Protein - FABP4) [50–52]. LD breakdown plays a crucial role in *C. burnetii* growth, as inhibiting host cell proteins involved in LD breakdown significantly impairs *C. burnetii* replication in macrophages [50]. Furthermore, inhibiting host DAG lipase, an enzyme that hydrolyzes diacylglycerol (DAG) into monoacylglycerol (MAG) and a free fatty acid, reduces *C. burnetii* growth [33]. DAG is a critical intermediate in lipid metabolism generated by LD breakdown, suggesting that LD-derived lipids may be required for *C. burnetii* intracellular growth and are therefore actively targeted by *C. burnetii* T4BSS effector proteins [33,50–52]. For example, *C. burnetii* phospholipases may either break down LDs or metabolize LD-derived lipids. Among putative phospholipases in the *C. burnetii* genome, only PldA (CBU0489) has been characterized. PldA remodels the bacterial membrane lipids during LCV to SCV transition [4]. However, more research is required to determine whether *C. burnetii*-derived phospholipases act on host cell LDs or LD-derived fatty acids or cholesterol serve as an energy source.

**Figure 2:**
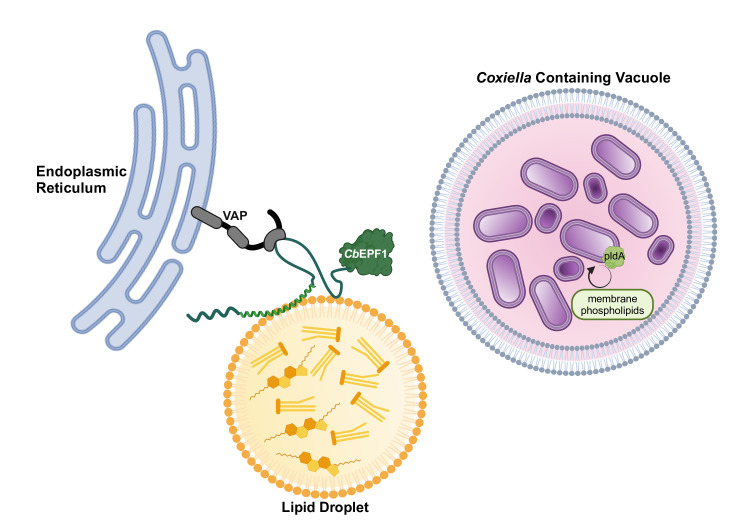
*C. burnetii* alters host lipid droplet metabolism through *Cb*EPF1. *C. burnetii* utilizes the T4BSS effector *Cb*EPF1 to modulate the formation and growth of lipid droplets. *Cb*EPF1-mediated LD-ER membrane contact sites are critical for regulating host lipid droplet size. Furthermore, *C. burnetii* phospholipase PldA may be involved in the breakdown of LD products. Created in BioRender. VanWinkle, P. (2024) https://BioRender.com/o87x987.

Thus far, only one *C. burnetii* T4BSS secreted effector protein is known to target LD metabolism directly. *C. burnetii* CBU1370, also known as *Cb*EPF1 (Effector Protein with FFAT motifs), induces LD biogenesis and growth [53]. Initially, *Cb*EPF1 localizes to the host ER at LD biogenesis sites and then relocates to the surface of growing LDs. At the LD surface, *Cb*EPF1 interacts with ER VAP proteins to establish ER-LD membrane contact sites and promote LD growth (Figure 2). As a result, *Cb*EPF1 expression leads to both an increase in both the size and number of LDs in the host cell [53]. LD-ER contact sites increase in *C. burnetii*-infected cells, further supporting a role for *Cb*EPF1 during infection. While the role of *Cb*EPF1 in pathogenesis is not known, one possibility is that *Cb*EPF1-mediated ER-LD contact sites, along with CCV-ER contact sites, reduce cholesterol toxicity to the bacteria by lowering CCV cholesterol levels. As discussed above, ORP1L is responsible for exporting CCV cholesterol at CCV-ER contact sites; in this context, *Cb*EPF1 may facilitate cholesterol packaging in ER-derived LDs.

### *C. burnetii* T4BSS effector proteins regulate CCV PIP species

**Figure 3:**
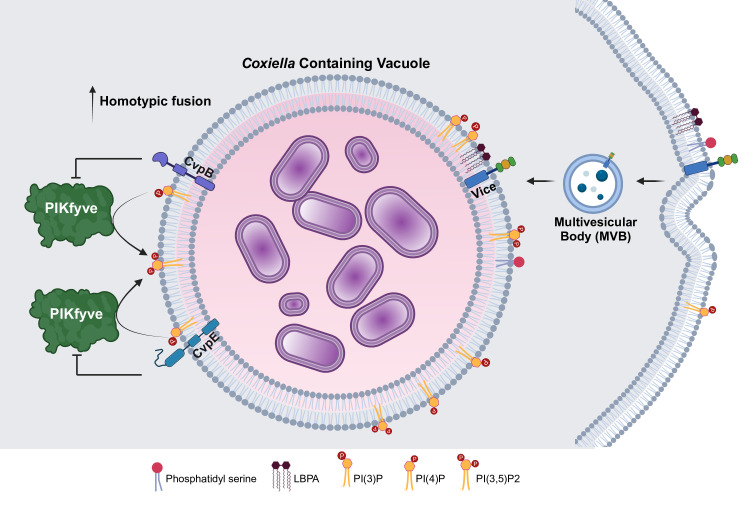
*C. burnetii* T4BSS effector proteins regulate CCV PIP species. *C. burnetii* T4BSS effector proteins CvpB, CvpE, and Vice modulate the recruitment or enrichment of different lipids and phosphoinositides on the CCV. Proteins CvpB and CvpE modulate PI3P phosphorylation by PIKfyve, while the effector Vice is involved in the recruitment of LBPA to CCV. Vice further promotes macropinocytosis required for CCV expansion. Regulated PIP levels on the CCV are critical for CCV homotypic fusion. Created in BioRender. VanWinkle, P. (2024) https://BioRender.com/e73y216.

Phosphatidylinositol phosphates (PIPs) on the membrane of bacterial-containing vacuole membranes act as protein docking sites, influencing signal transduction and vesicular trafficking events required for CCV expansion and maintenance. *C. burnetii* T4BSS effector proteins regulate PIP species on the CCV membrane, particularly PI(3)P and PI(4)P [23]. PI(3)P metabolism is the best studied, with the *C. burnetii* T4BSS effector proteins CvpB and CvpE shown to regulate host PI(3)P levels on the CCV (Figure 3) [23,24,28]. *C. burnetii* CvpB (also known as cig2/CBU0021) binds PI(3)P and phosphatidyl serine (PS) present on early endosomes and the CCV membrane [24]. CvpB binding to PI(3)P prevents the lipid kinase phosphoinositide 3-phosphate 5-kinase (PIKfyve) from converting PI(3)P to PI(3,5)P_2_. By stabilizing PI(3)P on the CCV, CvpB promotes autophagy-mediated CCV homotypic expansion. Although the role of homotypic fusion during infection is unclear, CvpB-deficient bacteria exhibit a multi-vacuolar phenotype without a significant replication defect in tissue culture [24,54]. However, CvpB may play a role during *in vivo* infection, as CvpB-deficient bacteria have a significant defect in colonization and survival in SCID mice [55]. *C. burnetii* CvpE (CBU1863) also binds PI(3)P and, similar to CvpB, is hypothesized to compete with PIKfyve for PI(3)P binding [28]. However, unlike CvpB, CvpE-mediated PI(3)P enrichment causes lysosomal enlargement by indirectly inhibiting TRPML1, a lysosomal Ca^+2^ channel. As a result, lysosomal fission is impaired, leading to enlarged lysosomes. Although it is not clear how this affects the CCV, CvpE-deficient bacteria are attenuated in both Vero cells and a SCID mouse model of infection [28].

The *C. burnetii* effector Vice (CBU2007) binds multiple PIP species as well as LBPA and is responsible for LBPA localization to the CCV [23]. Ectopic expression of Vice enhances macropinocytosis and the formation of large, CCV-like vesicles positive for PS, LBPA, and the monophosphorylated PIP species PI(3)P, PI(4)P, and PI(5)P. Vice increases vesicle stability by binding to and inhibiting the ESCRT complex, which is responsible for the formation of intraluminal vesicles in MVBs. Thus, Vice is a multi-functional effector required for CCV LBPA and is critical in regulating CCV membrane stability [23].

Elucidating how *C. burnetii* effector proteins modulate PIP species has the potential to uncover how the bacteria manipulate membrane dynamics to establish and maintain the CCV. However, because the motifs and domains involved in lipid binding in prokaryotes are poorly characterized, bioinformatic analysis may not be sufficient to discover additional T4BSS effector proteins involved in lipid binding and will require a careful biochemical approach. In addition to T4BSS effector proteins that directly bind PIP species, the *C. burnetii* genome also encodes several potential inositol-phosphate phosphatases (CBU0599, CBU0701, CBU1133). Although the function of these proteins remains unknown, they may regulate host cell PIP metabolism to impact various processes, including calcium release, gene expression, and cytoskeleton assembly [56]. This is true for other intracellular pathogens, including *Legionella pneumophila, C. burnetii’s* closest relative. *L. pneumophila* SidF is a secreted phosphatidylinositol polyphosphate 3-phosphatase that specifically dephosphorylates PI(3,4)P_2_ and PI(3,5)P_3_ to enrich PI(4)P-binding effectors on the *Legionella*-containing vacuole [57]. Similarly, *Salmonella* SopB is a secreted phosphoinositide phosphatase effector protein that recruits Rab5 and Vps34 and stimulates PI(3)P production on the *Salmonella*-containing vacuole [58]. The *sopB* deletion mutant displayed reduced intracellular growth, showing that its role in *Salmonella-*containing vacuole maturation is crucial for establishing a replicative niche in host cells [59]. This homology indicates the possibility that putative phosphatidylinositol phosphatases, as well as undiscovered *C. burnetii* T4BSS effector proteins, might benefit *C. burnetii* growth by promoting host protein recruitment via lipid modifications and direct interactions.

## Conclusion

Identifying the role of crucial host lipids in *C. burnetii* pathogenesis has marked a significant advancement in *C. burnetii* research over the last decade. For example, cholesterol has emerged as a crucial regulator of CCV pH, and PIPs play an essential role in CCV formation. Despite these insights, knowledge of the broader function of host lipids during *C. burnetii* infection remains limited. Whether *C. burnetii* can utilize host lipids as an energy source and the significance of specific lipids in the CCV membrane still need to be determined. Due to the inherent challenges in studying lipids, the dynamic recruitment of host lipids and their impact on CCV formation still needs to be explored. The *C. burnetii* secretome likely includes additional proteins that disrupt host lipid metabolism or act as lipid transporters, thereby altering host lipid homeostasis. Future advances in lipidomic techniques will facilitate the detection of lipid biomarkers and allow for a detailed analysis of changes in the host cell lipidome, down to molecular lipid species. Understanding how *C. burnetii* interferes with host lipid metabolism will provide new insights into host–pathogen interactions and may aid in developing more effective antibacterial therapies.

**Perspectives**

- *Coxiella burnetii* causes the disease Q fever and provides a robust model for understanding bacterial manipulation of host lipids.
- *Coxiella burnetii* has a diverse array of secreted effectors that modulate and sequester host lipids to maintain the *Coxiella* Containing Vacuole (CCV).
- Numerous putative lipid-modifying enzymes in the *Coxiella burnetii* genome represent interesting future research avenues that may facilitate improved understanding and treatment of *Coxiella burnetii* infection.

## Abbreviations

CCV: Coxiella Containing Vacuole
CE: cholesterol esters
DAG: diacylglycerol
ER: endoplasmic reticulum
25-HC: 25-hydroxycholesterol
HIV: Human immunodeficiency virus
LBPA: lysobisphosphatidic acid
LCV: large cell variant
LDs: Lipid droplets
MAG: monoacylglycerol
MVBs: multivesicular bodies
ORD: oxysterol binding domain
ORP1L: Oxysterol-Binding Protein-Related Protein 1 Long
PE: Phosphatidylethanolamine
PG: phosphatidylglycerol
PH: pleckstrin homology
PIP: Phosphatidylinositol phosphate
PS: phosphatidylserine
PX: phox homology
SCV: small cell variant
Stmp1: Sterol Modifying Protein-1
TAG: triacylglycerols
T4BSS: Type 4B Secretion System
